# Supplementary figures and images for: Treatment of corneal endothelial damage in a rabbit model with a bioengineered graft using human decellularized corneal lamina and cultured human corneal endothelium
Source: PLoS One. 2019 Nov 21;14(11):e0225480. doi: 10.1371/journal.pone.0225480 (PMC6871783; doi:10.1371/journal.pone.0225480)

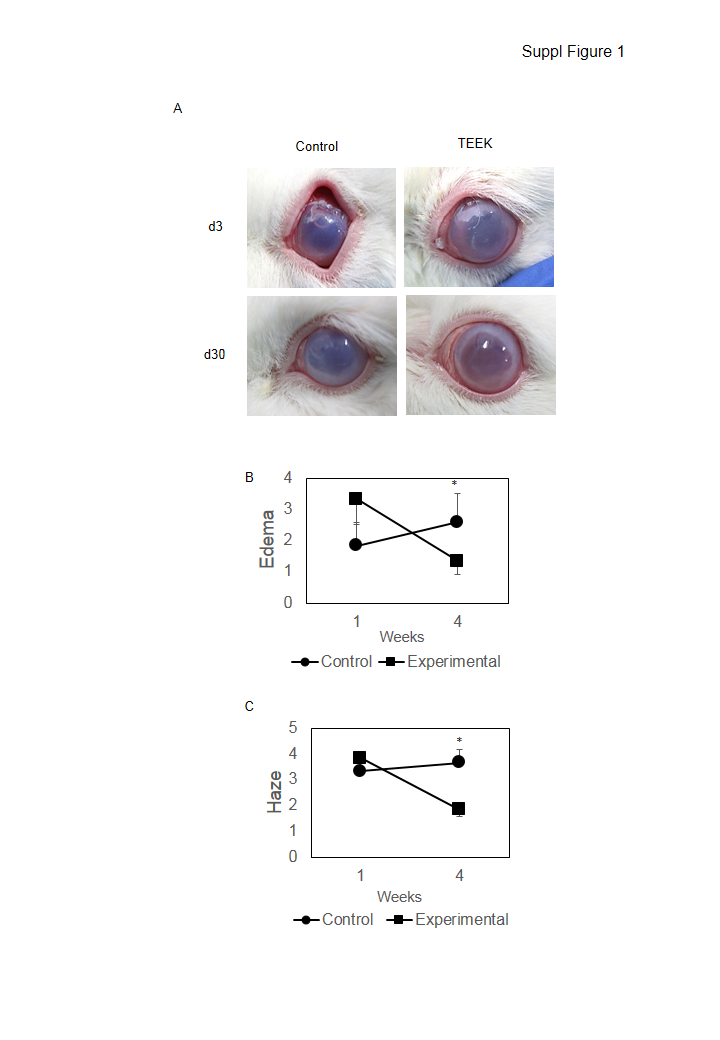

Supplement: S1 Fig — (A) Representative macroscopic live images of rabbit corneas subjected to lens removal and corneal endothelial damage 1 day and 4 weeks after surgery. Note the decreased haze in the experimental rabbit cornea at 4 weeks. Magnification 3x. (B and C) Progression of corneal oedema (B) and haze (C) graphs after 1 and 4 weeks. Data is shown as mean±SD. Asterisks indicate statistical significance at P ≤.05. (TIF) [file pone.0225480.s001.TIF]

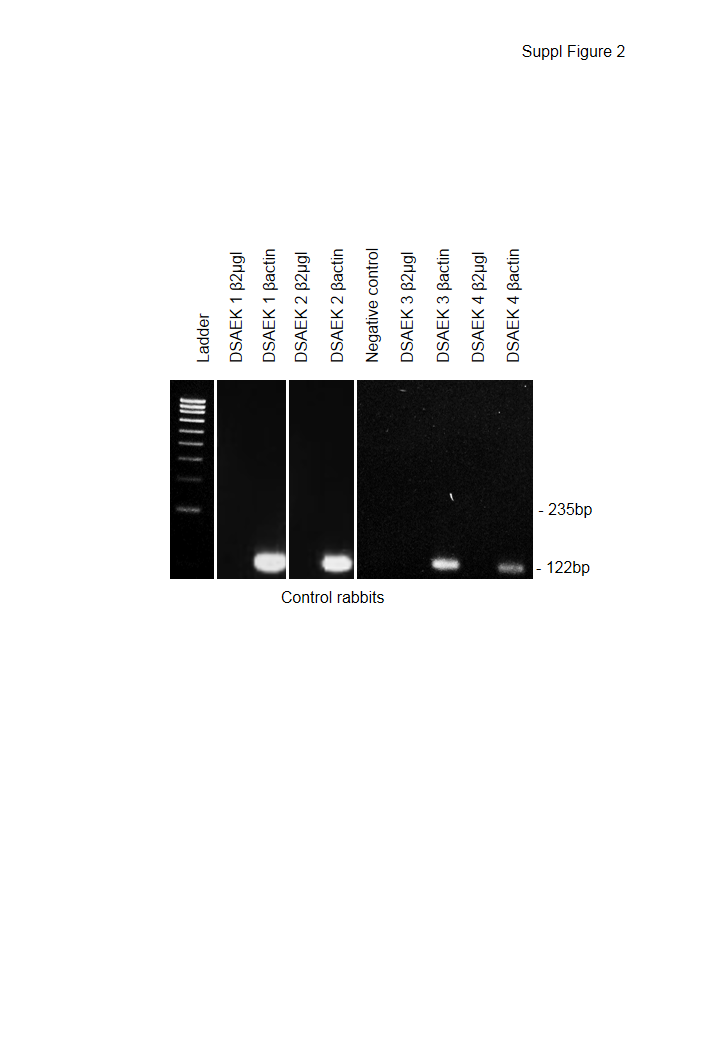

Supplement: S2 Fig — PCR amplification of the housekeeping gene β-actin at 122 bp and the human specific gene β2-microglobulin in control rabbits’ corneas. No control shows 235 bp β2-microglobulin amplification, whereas they do for the housekeeping β-actin gene. (TIF) [file pone.0225480.s002.TIF]
